# Early‐Life Betaine Supplementation Drives Sex‐Specific Metabolic Programming in Rats, Affecting FGF21 Signaling and Energy Balance

**DOI:** 10.1096/fj.202603380R

**Published:** 2026-10-16

**Authors:** Rocío A. Martín‐Chamorro, Catalina A. Pomar, Andreu Palou, Catalina Picó, Ana M. Rodríguez

**Affiliations:** ^1^ Laboratory of Molecular Biology, Nutrition and Biotechnology (Group of Nutrigenomics, Biomarkers and Risk Evaluation, NUBE) University of the Balearic Islands (UIB) Palma Spain; ^2^ Health Research Institute of the Balearic Islands (IdISBa) Palma Spain; ^3^ CIBER de Fisiopatología de la Obesidad y Nutrición (CIBEROBN) Madrid Spain; ^4^ Artificial Intelligence Research Institute of the Balearic Islands (IAIB) Palma Spain

**Keywords:** betaine, early‐life nutrition, FGF21 signaling, metabolic imprinting, sex‐specific metabolism

## Abstract

We aimed to evaluate the effects of maternal diet and betaine supplementation during the suckling period on *Fgf21* expression and metabolic pathways related to FGF21 signaling and metabolic programming. Offspring of Wistar rats fed either a chow diet (CD) or a Western diet (WD) were studied at postnatal day 22 and at 7 months. During suckling, pups received betaine at a physiological dose corresponding to twice the average intake estimated from milk consumption, or a vehicle. After weaning onto a CD, all animals were switched to a WD from months four to seven. Biometric parameters, circulating markers, and gene expression in hypothalamus, retroperitoneal white adipose tissue (rWAT), and liver were analyzed, together with cecal *Akkermansia muciniphila* abundance. At day 22, a maternal WD reduced hepatic *Fgf21* expression in both sexes; however, only males showed decreased *Klb* expression in rWAT, consistent with impaired FGF21 signaling. A maternal WD also reduced *A. muciniphila* abundance. Betaine increased *Fgfr1* expression in rWAT of female offspring from control dams. By seven months, betaine supplementation in males born to WD dams increased adiposity, and insulin and leptin levels, whereas females exhibited an improved plasma lipid profile (lower NEFA and TG). Hypothalamic *Drd2* and *Fgfr1* expression further showed sex‐specific modulation by the maternal diet and betaine. In conclusion, betaine supplementation during suckling exerts sex‐specific long‐term effects on metabolic programming. Females showed an improved lipid profile, whereas males, particularly those exposed to a maternal WD, developed increased adiposity and altered endocrine profiles. The findings suggest that early‐life betaine supplementation can modulate long‐term metabolic outcomes through sex‐specific effects on FGF21‐related pathways.

## Graphical abstract

A maternal western diet (WD) and betaine supplementation during suckling influence metabolic programming. A maternal WD induced adverse metabolic effects in offspring, accompanied by altered FGF21‐related gene expression and decreased *Akkermansia muciniphila* abundance. Betaine supplementation during suckling improved lipid profiles in females, but it increased adiposity and altered endocrine profiles in males exposed to a maternal WD. These sex‐specific effects were associated with differential hypothalamic *Drd2* and *Fgfr1* expression, suggesting that FGF21‐related pathways may contribute to the long‐term metabolic effects of early‐life betaine supplementation.

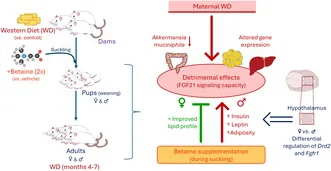

## 1 Introduction

Betaine, a naturally occurring methyl donor and osmolyte, plays a crucial role in one‐carbon metabolism by providing methyl groups for the synthesis of S‐adenosylmethionine (SAM), a universal methyl donor involved in epigenetic regulation [1]. Changes in one‐carbon metabolism during critical developmental windows have been associated with altered fetal growth trajectories, increased birth weight, and greater adiposity [2, 3, 4], suggesting that methyl donors, such as betaine, may contribute to early‐life metabolic programming.

The postnatal period, particularly lactation, represents a critical window of metabolic plasticity during which nutritional interventions can influence long‐term metabolic outcomes. Adverse maternal conditions, such as exposure to an obesogenic Western diet (WD), can predispose offspring to metabolic dysfunction [5, 6, 7] through alterations in metabolic and epigenetic pathways [8]. However, the lactation period may provide a key opportunity to counteract or alleviate these effects [9]. Betaine is naturally present in breast milk, and lower milk betaine concentrations have been associated with greater infant growth during early life [10], suggesting a potential role in metabolic programming.

Experimental studies further support a role for betaine in metabolic programming. Maternal betaine supplementation during lactation in mice has been associated with reduced adiposity and improved glucose homeostasis in adult male offspring, together with an increased abundance of *Akkermansia* spp. in the gut [10]. Similarly, maternal betaine intake during gestation and lactation has been shown to mitigate the metabolic consequences of maternal high‐fat diet (HFD) exposure in offspring, including non‐alcoholic fatty liver disease, potentially through modulation of the gut microbiota [11]. However, most studies have focused on maternal rather than direct postnatal supplementation of the offspring.

One key regulator of metabolic homeostasis that may mediate the effects of betaine is fibroblast growth factor 21 (FGF21), a liver‐derived hormone with an essential role in energy balance and metabolic regulation [12]. FGF21 signals through fibroblast growth factor receptors (FGFRs) in the presence of the co‐receptor β‐klotho in metabolic tissues and the central nervous system [12, 13]. In rodents, betaine supplementation increases hepatic and circulating FGF21 levels and is associated with improved glucose homeostasis, reduced hepatic lipid accumulation, and increased energy expenditure [14]. Importantly, some of these effects were not observed in *Fgf21* knockout mice, supporting a role for FGF21 in mediating the metabolic effects of betaine [14]. FGF21 also acts centrally to regulate nutrient intake, macronutrient preference, and energy expenditure through hypothalamic pathways [15], including effects on dopamine receptor 2 (D2R)‐expressing neurons [16].

Despite this evidence, it remains unclear whether betaine supplementation directly to pups during the suckling period can influence long‐term metabolic health, particularly in the context of maternal WD exposure. Furthermore, whether such effects are sex‐dependent and how they may influence hypothalamic regulation of energy homeostasis remains unknown. Therefore, this study aimed to investigate, in a rat model, the long‐term metabolic consequences of postnatal betaine supplementation during the suckling period in offspring of control‐ and WD‐fed dams, with a particular focus on FGF21 expression and metabolic pathways related to FGF21 signaling and metabolic programming.

## 2 Materials and Methods

### 2.1 Animals and Experimental Design

A diagram summarizing the experimental design is given in the Supporting Information. The experimental protocol received approval from the Bioethical Committee of the University of the Balearic Islands (Exp. 2018/13/AEXP, January 23, 2019) and was conducted following institutional guidelines. Part of the same cohort of animals (young rats) was used in a previous study, showing a key impact of the maternal WD on milk betaine (reduced), and milk miRNAs, and target genes in the adipose tissue of the offspring [17]. Briefly, virgin female Wistar rats were housed under controlled conditions and assigned to one of two dietary groups: one receiving a standard chow diet (CD, Altromin, Lage, Germany) (*n* = 10) and the other a high‐fat/high‐sucrose diet (WD, Research Diets, New Brunswick, NJ, USA) (*n* = 9). These dietary regimens began one month prior to mating and continued throughout pregnancy and lactation.

On postnatal day (PND) 1, litters were adjusted to 10 pups per dam, maintaining a balanced sex ratio (five males and five females when possible). Both male and female offspring received either betaine supplementation (Sigma Aldrich, Munich, Germany) (BET) or vehicle (water; VEH) via oral administration throughout the suckling period (PND1–PND20) using a pipette. The betaine dose was calculated to provide twice the average intake obtained through normal breastfeeding. To calculate betaine doses, we estimated rat pups' daily milk intake during lactation based on previous literature [18], along with the milk betaine concentration reported in a previous work from our lab [17]. Specifically, daily doses administered ranged from 6.5 to 160 μg throughout the suckling period (see Supporting Information).

Following weaning, at PND22, a subgroup of pups of each of the groups: male and female offspring of CD dams and WD dams, supplemented with VEH or BET during suckling (CD‐VEH, CD‐BET, WD‐VEH, and WD‐BET) was euthanized, while the remaining animals continued on a CD until 4 months of age. At this age, all the animals were transitioned to a WD as a metabolic challenge to unmask potential long‐term metabolic programming effects that may remain undetectable under standard diet conditions. Animals remained on the WD until 7 months of age, when they were euthanized. At both time points, blood, retroperitoneal white adipose tissue (rWAT), and the liver were collected for further analysis. The rWAT depot was selected as a representative visceral adipose tissue depot for gene expression analysis, based on previous evidence indicating that this depot may be particularly responsive to nutritional status compared with other adipose depots [19, 20]. Additionally, cecal fecal samples were collected at PND22, and the hypothalamus was obtained at 7 months of age. Inguinal WAT (iWAT) and interscapular brown adipose tissue (BAT) were also collected and weighed at 7 months of age. Body weight and food intake were monitored throughout the study. Body composition (fat and lean mass) was assessed using an EchoMRI‐700 (Echo Medical Systems LLC, Houston, TX, USA). The percentage of body fat was calculated as total adipose tissue weight divided by body weight × 100, and is reported in Figure 1 and Table 1 as a measure of adiposity. Blood glucose levels were determined from fresh samples using an Accu‐Chek glucometer (Roche Diagnostics, Mannheim, Germany). Further details on the nutritional composition of the diets are available in the Supporting Information.

**FIGURE 1:**
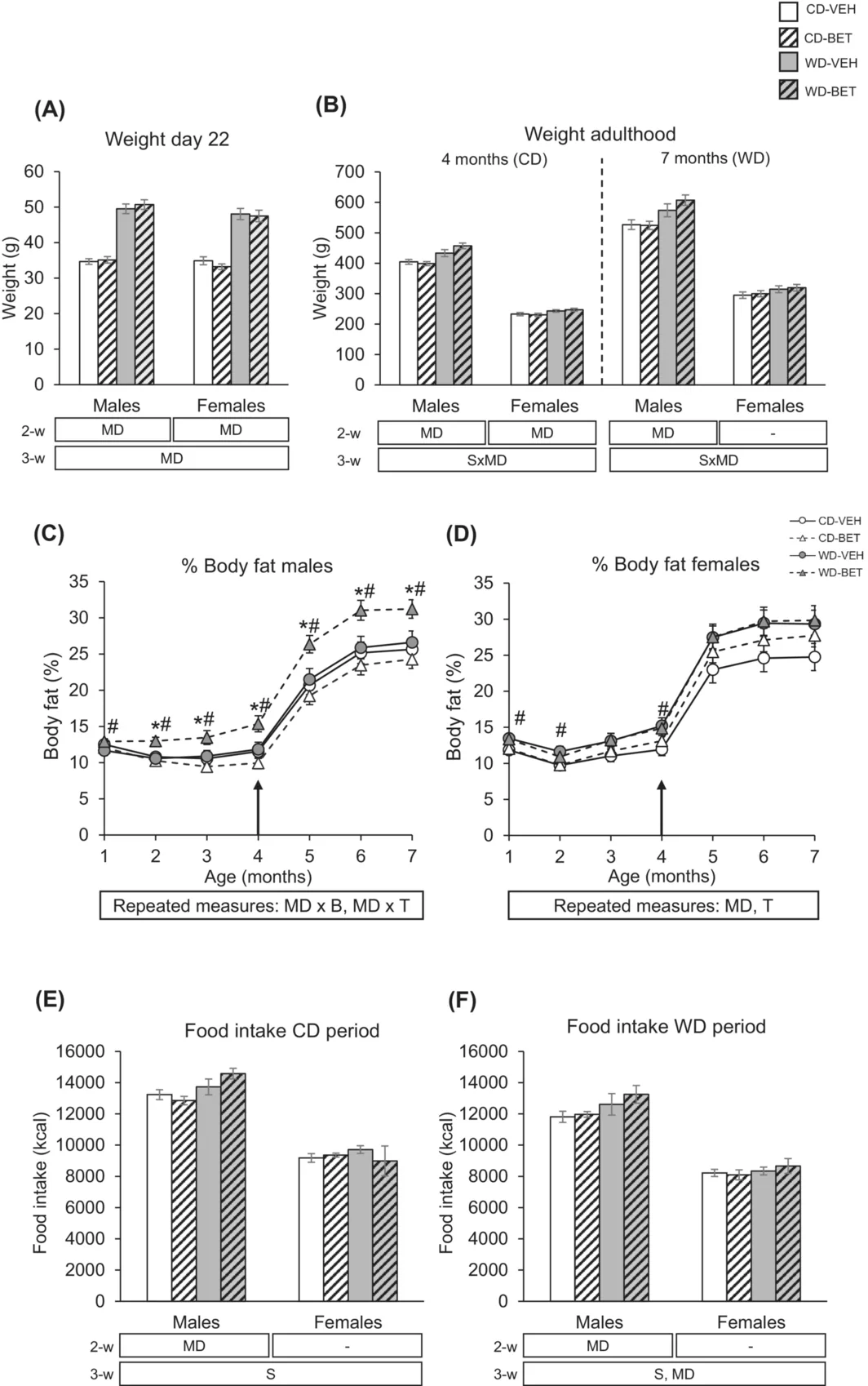
Body weight at postnatal day 22 (A) and at four and seven months of age (B); percentage of body fat from one to seven months of age in males (C) and females (D); and food intake during the control diet period (postnatal day 22 to four months of age) (E) and the WD period (four to seven months of age) (F). Data are presented as mean ± SEM. The arrows in figures C and D indicate the time point when the diet of the adult offspring was switched from control to a Western diet. For A, B, E, and F, a three‐way ANOVA was first performed with Sex (S), Maternal Diet (MD), and Betaine supplementation (B) as fixed factors; data stratified by Sex were used to run two‐way ANOVAs with MD and B as factors. In the two‐way ANOVA design, Tukey's HSD post hoc tests were applied exclusively to evaluate simple effects when a statistically significant interaction (MD × B) was detected. For C and D, data were analyzed using a mixed ANOVA stratified by sex, with MD and B as between‐subjects factors, and time (T) as a repeated‐measures factor, and sphericity violations were corrected using the Greenhouse–Geisser method; significant differences between groups across time points were determined via post hoc pairwise comparisons with Tukey's adjustment, where * indicates significant effects of betaine supplementation within the same dietary group and time point, while # indicates significant differences between maternal diet models within the same supplementation group and time point. Significance was set at *p* < 0.05. 2‐w, Two‐way ANOVA; 3‐w, Three‐way ANOVA; ANOVA, analysis of variance; BET, betaine treatment; CD, maternal control diet; T, time; VEH, vehicle treatment; WD, maternal Western diet.

**TABLE 1:** Body weight, liver weight, body fat weight, and weight of fat depots of male and female offspring at 7 months of age (*n* = 8–13).

			Males					Females					3‐way
			CD‐VEH	CD‐BET	WD‐VEH	WD‐BET	2‐way	CD‐VEH	CD‐BET	WD‐VEH	WD‐BET	2‐way	
7 m	Body weight (g)		527 ± 15.9	524 ± 13.5	574 ± 21.4	607 ± 17.0	MD	295 ± 10.5	300 ± 10.0	315 ± 11.4	320 ± 11.0	—	S × MD
	Liver weight (g)		15.0 ± 0.613	15.3 ± 0.490	17.0 ± 0.745	18.0 ± 0.849	MD	8.23 ± 0.319	8.30 ± 0.286	8.52 ± 0.295	8.74 ± 0.339	—	S × MD
	Body fat weight (g)		136 ± 10.6	127 ± 8.97	154 ± 13.4	189 ± 9.44 #	MD × B	74.3 ± 7.68	84.2 ± 7.10	93.6 ± 9.55	99.2 ± 10.3	—	S, MD
	Body fat (%)		25.7 ± 1.38	24.3 ± 1.28	26.6 ± 1.57	31.2 ± 1.28 #	MD × B	24.8 ± 1.88	27.7 ± 1.60	29.3 ± 1.92	29.9 ± 2.04	—	MD
	Fat depots	BAT (g)	0.873 ± 0.056	0.763 ± 0.049	0.815 ± 0.088	0.965 ± 0.070	—	0.519 ± 0.045	0.522 ± 0.050	0.549 ± 0.056	0.716 ± 0.048	MD	MD × B
		BAT (%)	0.695 ± 0.074	0.626 ± 0.048	0.537 ± 0.038	0.519 ± 0.036	MD	0.741 ± 0.071	0.653 ± 0.065	0.588 ± 0.029	0.766 ± 0.061	MD × B	S
		iWAT (g)	16.9 ± 1.64	14.4 ± 1.30	16.9 ± 1.67	19.9 ± 1.25	—	5.59 ± 0.534	6.29 ± 0.555	6.78 ± 0.763	6.84 ± 0.765	—	S, MD
		iWAT (%)	12.3 ± 0.320	11.2 ± 0.400	10.9 ± 0.270 #	10.4 ± 0.270	MD, B	7.63 ± 0.259	7.47 ± 0.195	7.23 ± 0.285	7.00 ± 0.413	—	S, MD, B
		rWAT (g)	21.5 ± 1.66	20.4 ± 1.46	25.0 ± 1.99	27.3 ± 1.67	MD	7.92 ± 0.964	8.13 ± 0.448	8.48 ± 1.01	8.52 ± 0.605	—	S × MD
		rWAT (%)	15.9 ± 0.540	16.1 ± 0.410	16.5 ± 0.760	14.7 ± 0.980	—	10.8 ± 0.640	10.1 ± 0.600	9.08 ± 0.550	9.06 ± 0.650	MD	S

*Note:* Body fat weight is reported in grams (g) and as a percentage (%) of body weight. Weights of specific fat depot are expressed in g and as a % of body fat weight. Data are mean ± SEM. Statistical analyses: A three‐way ANOVA was performed to assess the effects of sex (S), maternal diet (MD), and betaine treatment (B) (*p* < 0.05). A two‐way ANOVA was conducted to evaluate the effects of MD and B separately in males and females. Pairwise post hoc comparisons using Tukey's honest significant difference (HSD) test are given only when the two‐way ANOVA gives a statistically significant interaction (MD × B) (#, different from CD; *p* < 0.05).

Abbreviations: ANOVA, analysis of variance; BAT, brown adipose tissue; BET, betaine treatment; CD, maternal control diet; iWAT, inguinal white adipose tissue; rWAT, retroperitoneal white adipose tissue; VEH, vehicle treatment; WD, maternal Western diet.

### 2.2 Evaluation of Plasma Metabolic Parameters

Blood samples were collected and centrifuged (1000× *g*, 10 min, 4°C) to obtain plasma. Plasma metabolic parameters were analyzed in 7‐month‐old rats under *ad libitum* feeding conditions. Glucose, insulin, and Fibroblast growth factor 21 (FGF21) levels were also assessed under fasting conditions at 5.5 months of age. Insulin sensitivity was estimated using the Homeostatic Model Assessment for Insulin Resistance (HOMA‐IR), calculated from fasting glucose and insulin concentrations according to the following equation: HOMA‐IR = [fasting glucose (mmol·L^−1^) × fasting insulin (mU·L^−1^)]/22. Plasma triglycerides (TG), FGF21, nonesterified fatty acids (NEFA), and cholesterol were quantified using commercial enzymatic colorimetric kits, including the Serum Triglyceride kit (Sigma Diagnostics, St. Louis, MO, USA), Rat FGF‐21 ELISA Kit (Abcam, Cambridge, UK), NEFA‐HR Kit (Wako Chemicals GmbH, Neuss, Germany), and Cholesterol Kit (Biosystems SA, Barcelona, Spain), respectively. Additionally, insulin and leptin concentrations were determined through enzyme‐linked immunosorbent assay (ELISA) kits from Mercodia AB (Uppsala, Sweden) and R&D Systems (Minneapolis, MN, USA), respectively. Blood glucose levels were determined from fresh blood samples using an Accu‐Chek glucometer (Roche Diagnostics, Barcelona, Spain).

### 2.3 RNA Extraction

Total RNA was isolated from the liver and rWAT of 22‐day‐old and seven‐month‐old animals using the E.Z.N.A. Total RNA Kit I (Omega Bio‐Tek Inc., Norcross, GA, USA) following the manufacturer's protocol. Additionally, hypothalamic RNA from 7‐month‐old animals was extracted using the Total DNA, RNA, and Protein Isolation NucleoSpin Triprep Kit (Macherey‐Nagel, Düren, Germany). The RNA concentration and purity were subsequently assessed using a NanoDrop ND‐1000 spectrophotometer (NanoDrop Technologies Inc., Wilmington, DE, USA).

### 2.4 Real‐Time Quantitative Polymerase Chain Reaction (RT‐qPCR) Analysis

RT‐qPCR was performed to determine the mRNA expression levels of key metabolic and signaling genes in various tissues. In the liver, the expression of *Insr* (insulin receptor), *Fgf21* (fibroblast growth factor 21), *Irs1* (insulin receptor substrate 1), *Srebp1c* (sterol regulatory element‐binding protein 1c), and *Scd1* (stearoyl‐CoA desaturase 1) was analyzed. In rWAT, the expression of *Insr*, *Fgf21*, and *Irs1* was also measured, along with *Fgfr1* (fibroblast growth factor receptor 1), *Klb* (β‐klotho), *Pten* (phosphatase and tensin homolog), *Lep* (leptin), *Stat3* (signal transducer and activator of transcription 3), *Lpl* (lipoprotein lipase), *Adrb3* (β3‐adrenergic receptor), *Creb* (cAMP response element‐binding protein), and *Pnpla2* (patatin‐like phospholipase domain‐containing protein 2). In the hypothalamus, *Lepr* (leptin receptor), *Insr*, and *Fgfr1* expression levels were assessed, as well as the expression of genes involved in neuronal signaling and energy balance regulation, including *Drd2* (dopamine receptor D2), *Bdnf* (brain‐derived neurotrophic factor), *Prkaa2* (AMP‐activated protein kinase catalytic subunit α2), *Agrp* (agouti‐related peptide), *Npy* (neuropeptide Y), *Pomc* (pro‐opiomelanocortin), and *Cart* (cocaine‐ and amphetamine‐regulated transcript). *Gdi* (guanosine diphosphate dissociation inhibitor) was selected as the housekeeping gene.

### 2.5 Bacterial Profiling by q‐PCR in Cecal Feces

Total bacterial DNA was extracted from 200 mg of cecal feces obtained at PND22 using the E.Z.N.A. Stool DNA Kit (Omega Bio‐Tek Inc., Norcross, GA, USA) according to the manufacturer's protocol. The DNA concentration was measured using a NanoDrop ND‐1000 spectrophotometer (NanoDrop Technologies Inc., Wilmington, DE, USA). The relative abundance of *Akkermansia muciniphila* was assessed by quantifying 16S rRNA gene sequences through qPCR using specific primers. A universal primer targeting the conserved region of the 16S rRNA gene (total bacteria) was used as a reference to normalize bacterial DNA quantification, serving as a housekeeping gene for total bacterial content. Relative expression values were calculated in reference to the group of CD‐VEH male rats.

### 2.6 Statistical Analysis

Data are expressed as mean ± SEM. We first performed a three‐way ANOVA with sex (S), maternal diet (MD), and betaine supplementation (B) as fixed factors. Because we were specifically interested in sex‐specific responses, we then stratified the dataset by sex. For each sex, we ran a two‐way ANOVA with MD and B as the main factors. We show pairwise post hoc comparisons using Tukey's honest significant difference (HSD) test only when we detected a statistically significant interaction (MD × B). To evaluate changes in fat mass over time, we stratified data by sex and analyzed each group using a mixed ANOVA: the model included MD (Western vs. control) and B (vehicle or betaine supplementation) as between‐subjects factors, and T (time, monthly measurements) as a within‐subjects repeated‐measures factor; Greenhouse–Geisser corrections were automatically applied to the degrees of freedom whenever Mauchly's test indicated a violation of the sphericity assumption. We performed post hoc pairwise comparisons for MD and B effects across time points using Tukey's adjustment. We also conducted specific Pearson correlation analyses, reporting only statistically significant correlations. Statistical analyses were performed using RStudio version 2026.09.0 + 174 (Posit Software, PBC), with a significance threshold set at *p* < 0.05.

## 3 Results

### 3.1 Body Weight, Adiposity, and Food Intake

Body weight at PND22, and at four and seven months of age, as well as body fat percentage from one to seven months, and food intake during both the control diet period (PND22 to four months) and the Western diet period (four to seven months) of CD‐VEH, CD‐BET, WD‐VEH, and WD‐BET males and females are presented in Figure 1. The weights of the liver and fat depots at seven months are shown in Table 1. The effects of a maternal WD on offspring body weight were evident at PND22 in both males and females (Figure 1A). This difference persisted at four (in both sexes) and seven (only in males) months. Therefore, the most marked and significant increase in body weight in adulthood was observed in the WD‐BET group in males (Figure 1B).

When analyzing the body fat percentage over time, significant interactions between the maternal diet and betaine supplementation, and between the maternal diet and time, were observed in males (Figure 1C). Specifically, WD‐BET males displayed a higher body fat percentage from one to two months of age compared to the corresponding maternal diet‐ or betaine supplementation‐matched groups. In females, both the maternal diet and time affected body weight, but without any interaction (Figure 1D).

At seven months of age, considering both males and females, an effect of the WD was observed, resulting in higher body fat (even as a percentage) and, in males, higher body and liver weight (Table 1). Moreover, in males, body fat was especially increased by the combination of a maternal Western diet and betaine supplementation. Regarding adipose tissue depots, rWAT (in males) and iWAT (considering both sexes) weights were higher following maternal WD exposure. However, when iWAT was expressed as a percentage of total fat mass, both the maternal WD and betaine supplementation contributed to a reduction in this depot, an effect that was more marked and significant in males. In BAT, a significant interaction was observed between the maternal diet and betaine supplementation for BAT mass when both sexes were analyzed together. Upon sex stratification, a maternal WD significantly increased BAT mass in females. However, a maternal WD led to a lower BAT percentage in males. Notably, in females, there was an interactive MD × B effect in the percentage of BAT mass, given by the two‐way ANOVA, suggesting a specific modulatory effect of early‐life betaine supplementation in females when confronted with a maternal WD.

Regarding cumulative food intake, during the control diet period (PND22–4 months), maternal WD males consumed more calories than the control animals, independently of betaine supplementation (Figure 1E). During the WD period (4–7 months), a general maternal WD effect on food intake was observed considering both sexes, but it was only significant in males after sex stratification (Figure 1F); therefore, offspring of WD‐exposed mothers consumed more calories than those of CD‐exposed mothers, with the effect being more pronounced and significant in males.

### 3.2 Circulating Metabolic Parameters

Circulating parameters measured under *ad libitum* conditions in all male and female groups at 7 months of age are shown in Figure 2. Glucose, insulin, and FGF21 levels under fasting conditions at 5.5 months of age are also presented. Although the parameters measured at the time of sacrifice (7 months) and at 5.5 months of life (fasting parameters) might not be directly comparable (5.5 months is halfway through the Western diet challenge), this experimental design was necessary because the fasting procedure may induce metabolic stress in the animals. We, therefore, performed the fasting measurements sufficiently in advance of the final sacrifice to avoid potential interference with the collection of the main tissues and larger blood volumes required for the subsequent analyses.

**FIGURE 2:**
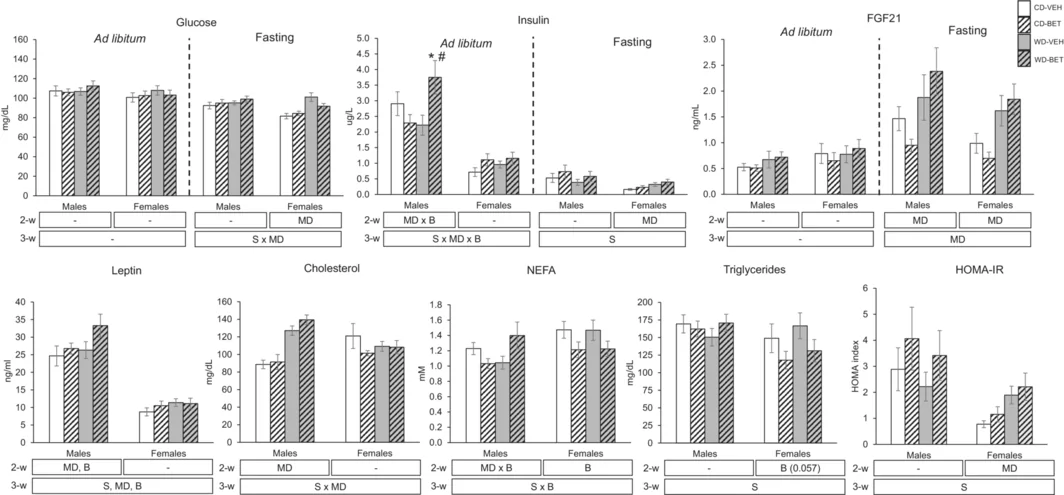
Circulating parameters in adult males and females under fed and/or fasting conditions (*n* = 8–13). Parameters assessed under fasting conditions (including HOMA‐IR) were measured at 5.5 months of age, whereas the other parameters shown in the figure as “ad libitum”, together with leptin, cholesterol, NEFA, and triglycerides, were measured at 7 months. Data are presented as mean ± SEM. Statistical analyses: A three‐way ANOVA was first performed with Sex (S), Maternal Diet (MD), and Betaine supplementation (B) as fixed factors; data stratified by Sex were used to run two‐way ANOVAs with MD and B as factors. In the two‐way ANOVA design, Tukey's HSD post hoc tests were applied exclusively to evaluate simple effects when a statistically significant interaction (MD × B) was detected (*, different from VEH; #, different from CD). Significance was set at *p* < 0.05 (except where indicated). 2‐w, Two‐way ANOVA; 3‐w, Three‐way ANOVA; ANOVA, analysis of variance; BET, betaine treatment; CD, maternal control diet; VEH, vehicle treatment; WD, maternal Western diet.

No significant differences in glucose levels were observed under *ad libitum* conditions. However, under fasting conditions, a maternal WD led to elevated circulating glucose levels in females. No significant differences were detected in males.

Regarding insulin levels under *ad libitum* conditions, betaine supplementation during the suckling period led to increased circulating insulin in male offspring of WD‐fed dams, with no effect observed in offspring of CD‐fed dams. In females, no significant effect of betaine was detected. Under fasting conditions, a maternal WD resulted in a significant increase in insulin levels in females, mirroring the rise observed in fasting glucose. Additionally, a maternal WD led to an increased HOMA index in females.

Maternal WD and BET supplementation during suckling significantly resulted in higher leptin levels, with this effect being more pronounced and statistically significant in males. Cholesterol concentrations were significantly increased in the male offspring of WD‐fed dams, regardless of betaine supplementation, whereas no significant changes were observed in females. In contrast, betaine supplementation during suckling significantly reduced circulating nonesterified fatty acids (NEFA) and tended to reduce triglycerides (TG) (*p* = 0.057) in females, regardless of maternal diet. A significant interaction between the maternal diet and betaine supplementation was observed for NEFA levels in males: betaine supplementation tended to lower NEFA levels in the SD group, whereas the opposite trend was observed in the WD group.

Under fasting conditions, a maternal WD had a pronounced effect, leading to elevated circulating FGF21 levels in both sexes. No significant differences were observed under ad libitum conditions.

### 3.3 Expression of *Fgf21* and Its Receptor/Co‐Receptor Signaling Complex in Liver and rWAT

Figure 3 summarizes the key changes in *Fgf21* signaling in the liver, its primary site of production, and in rWAT, which, although capable of secreting FGF21, primarily acts as a target tissue via *Fgfr1* and its co‐receptor *Klb* [12]. Gene expression was analyzed at both PND22 and seven months of age to assess the potential short‐ and long‐term metabolic programming effects of the maternal diet and betaine supplementation during suckling.

**FIGURE 3:**
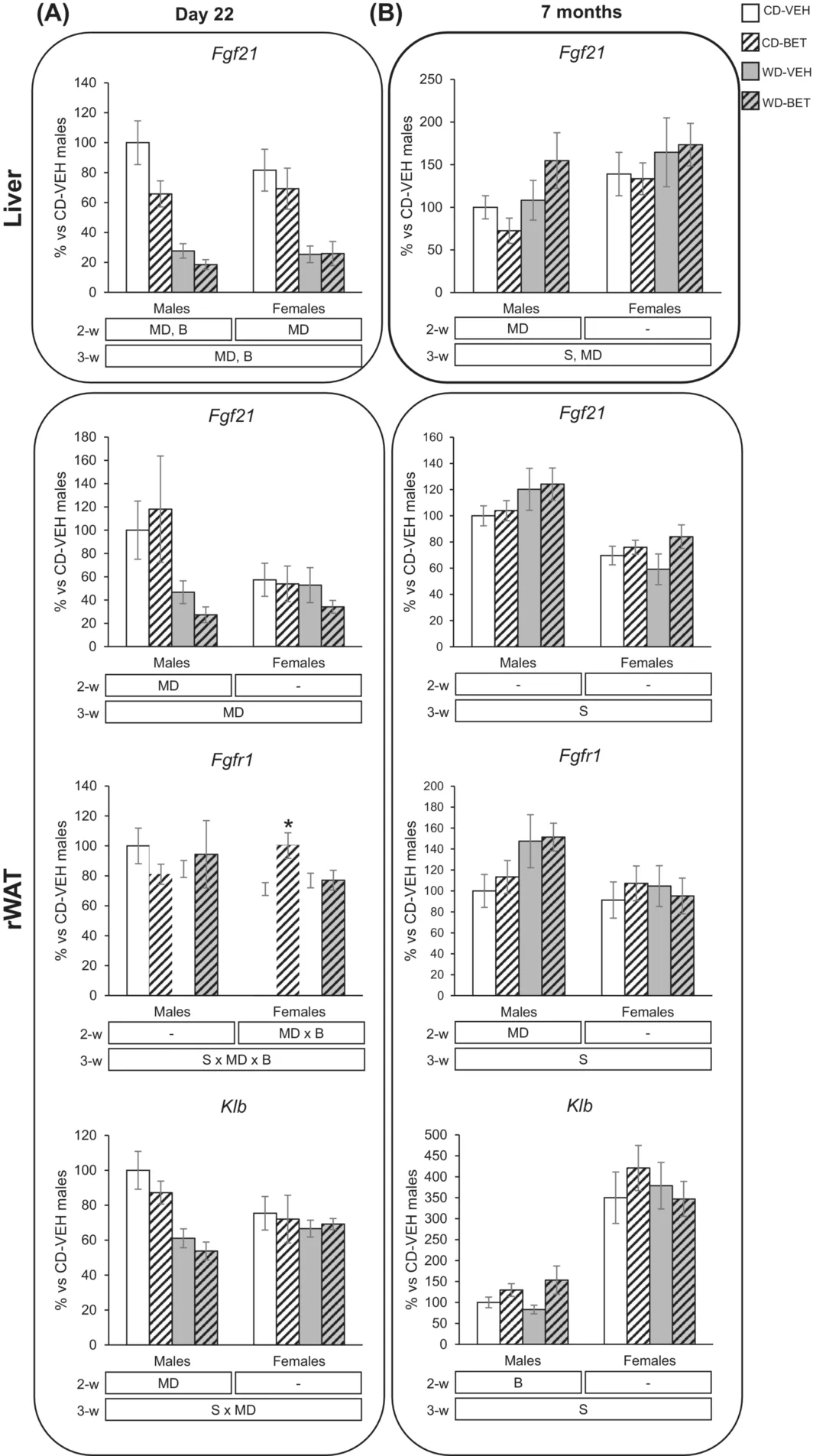
FGF21 signaling in the liver and rWAT on postnatal day 22 (A) and at seven months of age (B). Gene expression is presented as a percentage relative to the CD‐VEH male group. Data are shown as mean ± SEM. Statistical analyses: A three‐way ANOVA was first performed with Sex (S), Maternal Diet (MD), and Betaine supplementation (B) as fixed factors; data stratified by Sex were used to run two‐way ANOVAs with MD and B as factors. In the two‐way ANOVA design, Tukey's HSD post hoc tests were applied exclusively to evaluate simple effects when a statistically significant interaction (MD × B) was detected (*, different from VEH). Significance was set at *p* < 0.05. 2‐w, Two‐way ANOVA; 3‐w, Three‐way ANOVA; ANOVA, analysis of variance; BET, betaine treatment; CD, maternal control diet; *Fgf21*, fibroblast growth factor 21; *Fgfr1*, fibroblast growth factor receptor 1; *Klb*, β‐klotho; VEH, vehicle treatment; WD, maternal Western diet.

At PND22 (Figure 3A), a significant downregulation of hepatic *Fgf21* expression was observed in both male and female offspring of dams exposed to a WD. Additionally, betaine supplementation during suckling led to lower *Fgf21* expression levels in males but not in females. In rWAT, a maternal WD also resulted in significantly lower *Fgf21* expression, especially in males. Regarding FGF21 signaling components, a maternal WD did not affect *Fgfr1* expression levels but resulted in lower *Klb* expression in males. Notably, betaine supplementation increased *Fgfr1* expression in females born to CD‐fed dams but had no effect in the offspring of WD‐exposed dams.

By seven months of age (Figure 3B), pronounced sex differences were evident in hepatic and rWAT *Fgf21* expression, as well as in *Fgfr1* and *Klb* levels in rWAT. In the liver, *Fgf21* expression was increased in the offspring of dams exposed to WD, with the effect being significant in males. A maternal WD did not result in significant changes in *Fgf21* expression in rWAT. Additionally, *Fgfr1* expression levels increased in males due to the maternal WD, while *Klb* expression was greater due to betaine supplementation, only in males.

### 3.4 Expression of Energy and Lipid Metabolism‐Related Genes in Liver and rWAT

To further characterize the effects of betaine supplementation during suckling and maternal diet during the perinatal period, we examined the expression of key genes involved in energy and lipid metabolism in the liver and rWAT at 7 months of age.

In the liver (Figure 4A), betaine supplementation during suckling resulted in lower expression levels of *Irs1* and the lipogenesis‐related gene *Srebp1*, an effect especially evident in females. Additionally, a maternal WD downregulated *Scd1* expression exclusively in males. No significant differences were observed between groups in the expression levels of *Insr*, although females exhibited higher expression levels than males.

**FIGURE 4:**
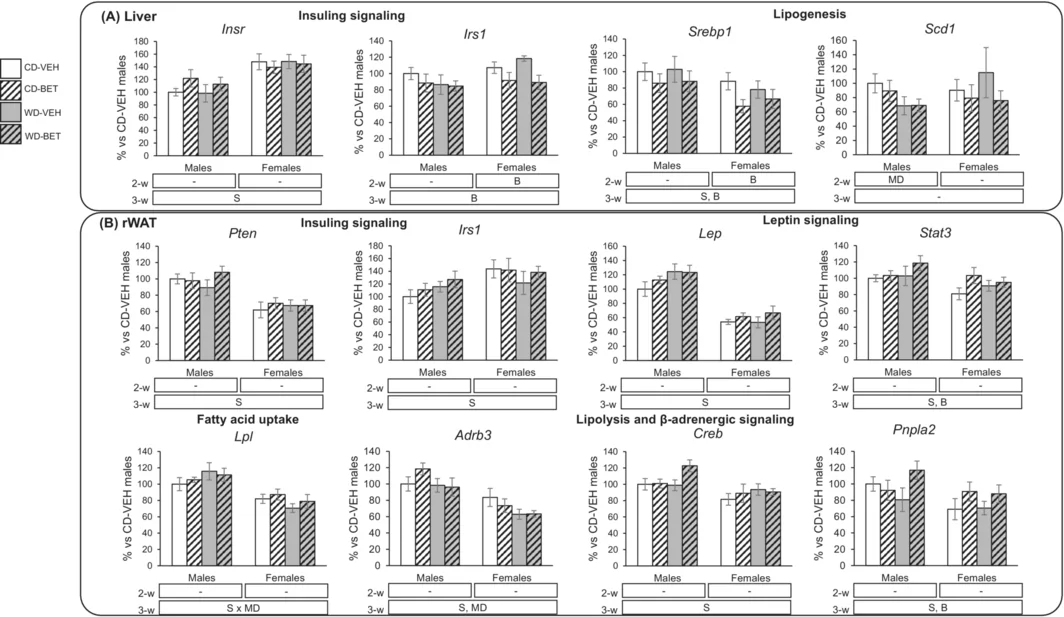
Gene expression in the liver and rWAT at seven months of age. (A) Insulin signaling pathway in the liver; Lipogenesis in the liver. (B) Insulin signaling pathway in rWAT; Leptin signaling in rWAT; Fatty acid uptake in rWAT; Lipolysis and β‐adrenergic signaling in rWAT. Gene expression levels are presented as a percentage relative to the CD‐VEH male group. Data are shown as mean ± SEM. Statistical analyses: A three‐way ANOVA was first performed with Sex (S), Maternal Diet (MD), and Betaine supplementation (B) as fixed factors; data stratified by Sex were used to run two‐way ANOVAs with MD and B as factors. In the two‐way ANOVA design, Tukey's HSD post hoc tests were applied exclusively to evaluate simple effects when a statistically significant interaction (MD × B) was detected. Significance was set at *p* < 0.05. 2‐w, Two‐way ANOVA; 3‐w, Three‐way ANOVA; ANOVA, analysis of variance; BET, betaine treatment; CD, maternal control diet; WD, maternal Western diet; *Insr*, insulin receptor; *Irs1*, insulin receptor substrate 1; *Srebp1*, sterol regulatory element‐binding protein 1; *Scd1*, stearoyl‐CoA desaturase 1; *Pten*, phosphatase and tensin homolog; *Lep*, leptin; *Stat3*, signal transducer and activator of transcription 3; *Lpl*, lipoprotein lipase; *Adrb3*, β3‐adrenergic receptor; *Creb*, cAMP response element‐binding protein; *Pnpla2*, patatin‐like phospholipase domain‐containing protein 2; VEH, vehicle treatment.

In rWAT (Figure 4B), no significant differences were found in the expression levels of most of the insulin and leptin signaling‐related genes studied due to the maternal diet or betaine supplementation during suckling, particularly after stratifying by sex. However, the expression levels of *Stat3*, when analyzing both sexes together, were significantly increased by betaine supplementation during the suckling period. Regarding genes involved in adrenergic activation and lipolysis, A*drb3* expression levels were decreased due to maternal WD exposure, whereas *Pnpla2* expression was upregulated by betaine supplementation. Additionally, sex‐associated differences were observed regardless of treatment, with females showing lower expression levels of *Pten*, *Lep*, *Stat3*, *Creb*, and *Pnpla2*, and higher expression of *Irs1* compared to males.

### 3.5 Expression of Appetite Regulation and Neuroendocrine Signaling‐Related Genes in the Hypothalamus

Expression levels of selected energy control and FGF21 signaling genes in the hypothalamus were studied at seven months of age (Figure 5). The expression of *Drd2* and *Fgfr1* was significantly upregulated due to betaine supplementation in females (regardless of maternal diet) and in the male offspring of WD‐fed dams. Additionally, *Prkaa2* expression levels were significantly reduced by a maternal WD (especially in males) and by betaine supplementation (particularly in females), and *Insr* expression levels were reduced by betaine supplementation in females.

**FIGURE 5:**
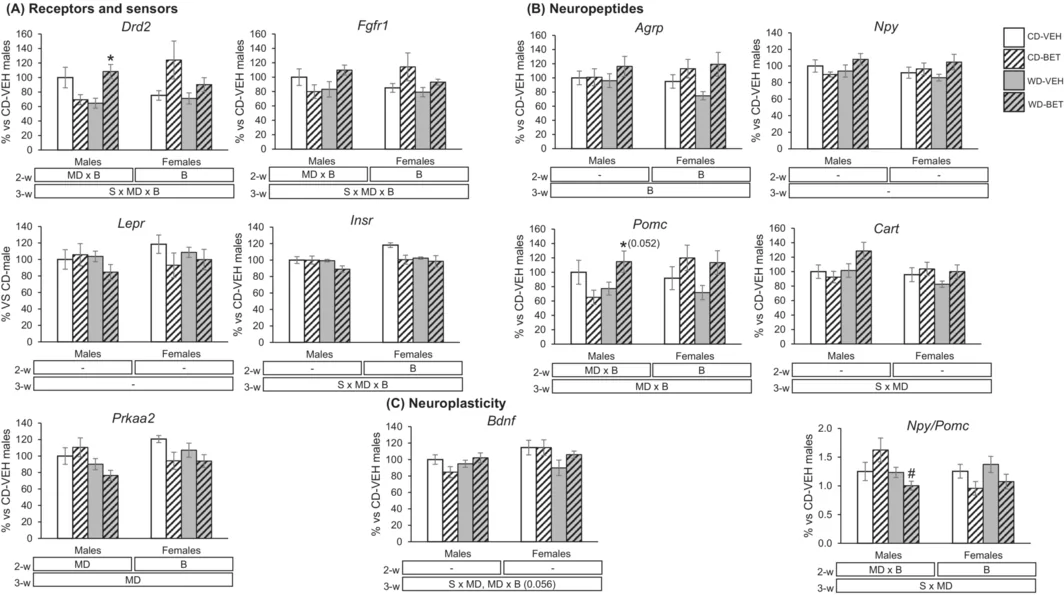
Hypothalamic gene expression at seven months of age. (A) Receptors and sensors. (B) Neuropeptides. (C) Neuroplasticity‐related genes. Gene expression levels are presented as a percentage relative to the CD‐VEH male group. Data are shown as mean ± SEM. Statistical analyses: A three‐way ANOVA was first performed with Sex (S), Maternal Diet (MD), and Betaine supplementation (B) as fixed factors; data stratified by Sex were used to run two‐way ANOVAs with MD and B as factors. In the two‐way ANOVA design, Tukey's HSD post hoc tests were applied exclusively to evaluate simple effects when a statistically significant interaction (MD × B) was detected (*, different from VEH). Significance was set at *p* < 0.05 (except where indicated). 2‐w, Two‐way ANOVA; 3‐w, Three‐way ANOVA; *Agrp*, agouti‐related peptide; ANOVA, analysis of variance; BET, betaine treatment; *Bdnf*, brain‐derived neurotrophic factor; *Cart*, cocaine‐ and amphetamine‐regulated transcript; CD, maternal control diet; *Drd2*, dopamine receptor D2; *Fgfr1*, fibroblast growth factor receptor 1; *Lepr*, leptin receptor; *Insr*, insulin receptor; *Npy*, neuropeptide Y; *Pomc*, pro‐opiomelanocortin; *Prkaa2*, AMP‐activated protein kinase catalytic subunit α2; VEH, vehicle treatment; WD, maternal Western diet.

Regarding hypothalamic neuropeptides, betaine supplementation during the suckling period resulted in higher expression levels of *Agrp* (with a more pronounced and significant effect in females) and *Pomc* in females and the male offspring of dams exposed to a WD. *Npy* showed no changes by the ANOVA analysis, and *Cart* expression levels showed an interactive S × MD effect due to the tendency toward higher levels in males of the WD‐BET group. Notably, the *Npy/Pomc* ratio, a key indicator of the orexigenic/anorexigenic balance [21], was significantly decreased by betaine supplementation in females, regardless of maternal diet, and in WD‐BET males compared to CD‐BET males.

No significant differences between groups were observed in *Bdnf* expression, but an interactive effect was found between sex and maternal diet, and a tendency (*p* = 0.056) for maternal diet and betaine supplementation interaction. No significant differences were found in the expression of *Lepr* between groups.

### 3.6 Cecal *Akkermansia muciniphila* Abundance

Figure 6 illustrates the relative abundance of *A. muciniphila* in cecal feces at PND22. Overall, *A. muciniphila* abundance was significantly reduced in response to a maternal WD.

**FIGURE 6:**
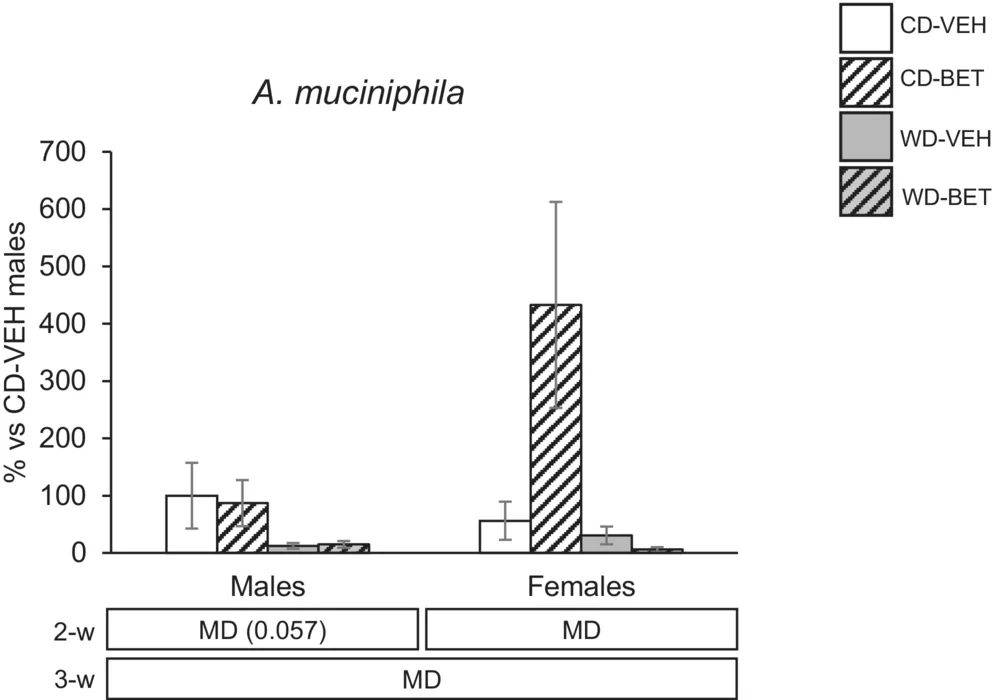
Relative DNA abundance of *A. muciniphila* in cecal feces at postnatal day 22. The relative abundance is expressed as a percentage relative to the CD‐VEH male group. Data are presented as mean ± SEM. Statistical analyses: A three‐way ANOVA was first performed with Sex (S), Maternal Diet (MD), and Betaine supplementation (B) as fixed factors; data stratified by Sex were used to run two‐way ANOVAs with MD and B as factors. In the two‐way ANOVA design, Tukey's HSD post hoc tests were applied exclusively to evaluate simple effects when a statistically significant interaction (MD × B) was detected. Significance was set at *p* < 0.05 (except where indicated). 2‐w, Two‐way ANOVA; 3‐w, Three‐way ANOVA; *A. muciniphila* , *Akkermansia muciniphila*; ANOVA, analysis of variance; BET, betaine treatment; CD, maternal control diet; VEH, vehicle treatment; WD, maternal Western diet.

## 4 Discussion

Previous studies have linked maternal betaine status during gestation and lactation to offspring body weight and adiposity [4, 10]. While most animal studies report beneficial effects of maternal betaine supplementation during the perinatal period [10, 11, 14, 22, 23], the long‐term effects of direct betaine supplementation to offspring during suckling remain unexplored. Here, we investigated, in rats, the effects of betaine supplementation during this period in offspring born to control dams and to dams exposed to a WD before mating and throughout gestation and lactation, with all offspring subsequently challenged with a WD in adulthood. Notably, WD‐exposed dams have been previously shown to have markedly lower betaine levels in milk (approximately 80% lower), potentially limiting its transfer to the offspring [5, 17]. This provides a rationale for investigating betaine supplementation during suckling.

The maternal WD and betaine supplementation interacted to shape offspring metabolic phenotypes in a sex‐dependent manner. In adult females exposed to a WD, betaine supplementation improved the lipid profile, with lower circulating NEFAs and a tendency toward lower TG levels, consistent with previous reports of reduced NEFAs following maternal betaine supplementation [23]. In contrast, males in the WD‐BET group showed a progressive increase in body fat percentage from early life to adulthood, suggesting that, rather than counteracting maternal WD effects, direct betaine supplementation during suckling may exacerbate them in males. Similarly, maternal methyl donor supplementation during lactation, including betaine, combined with maternal HFD exposure, increased fat mass in male offspring reexposed to an HFD in adulthood [24]. This contrasts with other studies linking maternal betaine supplementation to reduced adiposity and improved metabolic outcomes [22]. These discrepancies may stem from differences in the mode (maternal or offspring) and timing of supplementation, as well as interactions with the maternal diet.

The maternal WD and betaine supplementation also affected circulating metabolic markers. WD‐BET males had higher insulin levels than their vehicle‐treated counterparts, while leptin levels were increased by both the maternal WD and betaine supplementation in both sexes, with a greater effect in males. These findings are consistent with evidence that maternal HFD exposure disrupts hypothalamic leptin signaling and energy homeostasis in offspring [25], an effect that may be exacerbated by betaine supplementation in males.

FGF21 has been proposed as a potential mediator of the metabolic effects of betaine [14, 26, 27]. FGF21 responsiveness is closely linked to metabolic homeostasis, and reduced *Klb* expression in adipose tissue has been associated with greater susceptibility to diet‐induced metabolic dysfunction [28]. Therefore, maintaining *Klb* and *Fgfr1* expression in adipose tissue under metabolic stress seems crucial for preserving FGF21 sensitivity. Preclinical studies suggest that females retain greater sensitivity to FGF21, with HFD‐fed females upregulating *Fgfr1*, *Fgfr2*, and *Klb*, whereas males appear more susceptible to FGF21 resistance [27]. In our study, maternal WD reduced hepatic *Fgf21* expression at PND22 in both sexes, whereas only males showed decreased *Klb* expression in rWAT. Females maintained *Klb* expression, and, among offspring of control dams, betaine supplementation increased *Fgfr1* expression. These findings suggest sex‐specific differences in adipose FGF21‐related signaling that may contribute to the distinct long‐term metabolic responses to betaine.

Differences in *Fgf21* expression between PND22 and adulthood further suggest age‐dependent changes in its metabolic regulation. FGF21 levels have been reported to be decreased in obese children and negatively correlated with insulin, HOMA‐IR, and leptin levels [29]. Consistently, in our study, hepatic *Fgf21* expression at PND22 was negatively correlated with adiposity and circulating leptin levels (Supporting Information). In adulthood, however, offspring of WD‐exposed dams exhibited increased fasting FGF21 levels and hepatic *Fgf21* expression levels, particularly in males. This transition from lower early‐life expression to adult upregulation may reflect a compensatory response to chronic metabolic dysregulation. Similar patterns have been reported in obesity models, where chronic metabolic stress promotes a transition from FGF21 sensitivity to resistance, ultimately impairing its regulatory effectiveness [30, 31]. This maladaptive response may be especially significant upon reexposure to a WD in adulthood, further exacerbating metabolic dysfunction.

Regarding other genes involved in energy metabolism, betaine supplementation during suckling was associated with a general downregulation of hepatic *Srebp1* (especially in females), which may contribute to a healthier lipid profile. Accordingly, hepatic *Srebp1* and *Scd1* mRNA levels were positively correlated with plasma TG levels (Supporting Information), consistent with previous evidence that betaine suppresses *Srebp1c* expression, thereby reducing hepatic lipogenesis and fat accumulation [32]. In males, reduced *Scd1* expression in offspring of WD‐fed dams may reflect long‐term metabolic programming (given by the maternal WD diet) rather than an acute dietary response, as all groups were maintained on a WD. In rWAT, betaine supplementation increased *Stat3* expression across sexes and maternal diets, suggesting modulation of the leptin–STAT3 axis. Conversely, reduced *Adrb3* expression may indicate impaired adrenergic signaling capacity in the rWAT associated with maternal WD exposure, potentially affecting lipolysis and energy expenditure [33]. Meanwhile, the increased *Pnpla2* expression in both sexes following betaine supplementation during suckling may indicate enhanced lipolysis capacity, thereby contributing to improved metabolic flexibility.

FGF21 can cross the blood–brain barrier and activate hypothalamic GABAergic neurons expressing dopamine receptor 2 (D2R, *Drd2*), contributing to the regulation of food intake and energy expenditure [16, 34]. In this context, hypothalamic *Drd2* and *Fgfr1* expression patterns observed here suggest that perinatal betaine supplementation modulates FGF21 signaling—again, in a sex‐specific manner. Both genes were upregulated in betaine‐supplemented females and in males exposed to maternal WD and supplemented with betaine. In females, this may reflect enhanced sensitivity of the FGF21‐D2R system in GABA‐containing neurons, potentially contributing to their more favorable metabolic profile. However, this pattern did not translate into metabolic protection in WD‐exposed males, who also showed increased *Drd2* and *Fgfr1* expression by betaine supplementation but developed greater adiposity and metabolic dysfunction. Thus, hypothalamic upregulation of these genes may not be sufficient to prevent the long‐term effects of a maternal WD.

Early‐life metabolic programming following maternal WD exposure can influence appetite regulation [35]. In our study, offspring of WD‐fed dams consumed more calories, with the effect being particularly evident in males, who showed increased food intake over time. Hypothalamic gene expression at seven months only partially explains these differences. Although betaine supplementation was generally associated with a lower *Npy*/*Pomc* mRNA ratio, indicative of reduced orexigenic drive [21], this pattern did not prevent hyperphagia in males exposed to a maternal WD.

FGF21 signaling has also been associated with increased total caloric intake in rodents [36], potentially through hypothalamic effects on orexigenic pathways [36, 37]. In our study, although no changes were found in *Npy* expression, males from the WD‐BET group showed increased *Drd2* and *Fgfr1* expression, potentially indicating enhanced central FGF21 responsiveness, particularly under conditions of elevated circulating FGF21. Conversely, increased *Pomc* expression in this group may reflect a compensatory response, possibly linked to elevated leptin levels. Interestingly, *Drd2* and *Fgfr1* expression were strongly correlated across all groups, and the expression of both genes also correlated positively with *Agrp*, *Npy*, *Pomc*, and *Cart* expression (Supporting Information).

Previous studies in mice have suggested that the metabolic effects of maternal betaine supplementation may involve modulation of the gut microbiota, including an increased *Akkermansia* abundance in offspring [10]. *Akkermansia* has attracted considerable interest due to its inverse association with obesity and metabolic disorders in humans and animal models [38, 39], and in vitro studies further support a direct role for betaine in promoting the growth of *A. muciniphila* [10]. In contrast, in our study, maternal WD reduced *A. muciniphila* abundance at PND22 in both sexes, regardless of betaine supplementation. Thus, direct betaine supplementation during suckling did not reproduce the microbial effects reported following maternal supplementation. This difference may reflect the distinct timing and route of supplementation, as well as interactions with the maternal diet and sex, highlighting the context‐dependent effects of betaine on the early‐life gut microbiota.

Overall, our findings highlight the sex‐ and context‐dependent effects of early‐life betaine supplementation. Betaine supplementation during suckling was associated with an improved lipid profile in females, whereas in males exposed to a maternal WD, it was associated with increased adiposity and altered metabolic profiles. These sex‐specific outcomes were accompanied by differential changes in hypothalamic *Drd2* and *Fgfr1* expression, suggesting that FGF21‐related pathways may contribute to the long‐term metabolic effects of early‐life betaine supplementation. Further studies are needed to clarify the mechanisms underlying these sex‐specific responses.

## Author Contributions

A.P., C.P., and A.M.R. designed research. R.A.M.‐C., and C.A.P. conducted research. R.A.M.‐C. and A.M.R. analyzed data. R.A.M.‐C., C.A.P., A.P., C.P., and A.M.R. participated in the discussion of the results. R.A.M.‐C. and A.M.R. drafted the first version of the manuscript. R.A.M.‐C., C.A.P., A.P., C.P., and A.M.R. revised and corrected the final version of the manuscript. All authors approved the final manuscript.

## Funding

Spanish Government, Grant/Award Numbers: PGC2018‐097436‐B‐I00 and PID2022‐138140NBI00 (funded by MCIN/AEI/10.13039/501100011033 and by “ERDF A way of making Europe”).

## Conflicts of Interest

The authors declare no conflicts of interest.

## Supporting information


**Figure S1:** Experimental design. The experiment followed both male and female offspring. The betaine supplementation was equivalent to twice (2×) the average intake obtained during breastfeeding. Animal distribution: CD‐dams (*n* = 12), WD‐dams (*n* = 12); short‐term offspring CD‐VEH (12 males, 10 females), CD‐BET (13 males, 12 females), WD‐VEH (10 males, 10 females), and WD‐BET (8 males, 9 females); long‐term offspring CD‐VEH (14 males, 12 females), CD‐BET (14 males, 12 females), WD‐VEH (11 males, 12 females), and WD‐BET (13 males, 12 females). C, control; BET, betaine; PND, postnatal day; VEH, vehicle; WD, Western diet.
**Figure S2:** Hepatic *Fgf21* mRNA levels at postnatal day 22 are negatively correlated with adiposity in offspring. The scatter plot depicts the negative correlation between hepatic *Fgf21* mRNA expression (normalized to housekeeping genes) and body fat percentage in offspring at postnatal day 22. Each dot represents an individual animal. Pearson's correlation coefficient (*R*) and significance (*p*‐value) are indicated on the plot.
**Figure S3:** Hepatic *Fgf21* mRNA levels at postnatal day 22 are negatively correlated with circulating leptin levels in offspring. The scatter plot illustrates the negative correlation between hepatic *Fgf21* mRNA expression (normalized to housekeeping genes) and circulating leptin levels in offspring at postnatal day 22. Each dot represents an individual animal. Pearson's correlation coefficient (*R*) and significance (*p*‐value) are indicated on the plot.
**Figure S4:** The hepatic expression of *Srebp1* and *Scd1* is positively correlated with circulating triglyceride (TG) levels in offspring at 7 months of age. The scatter plots show the positive correlation between circulating TG levels and hepatic mRNA expression (normalized to housekeeping genes) of Srebp1 (A) or Scd1 (B) in offspring at 7 months of age. Each dot represents an individual animal. Pearson's correlation coefficient (*R*) and significance (*p*‐value) are shown on each plot.
**Table S1:** Correlation between hypothalamic gene expression levels. The table shows the correlations between *Drd2* and *Fgfr1* expression (mRNA), as well as their correlations with the expression of neuropeptides involved in energy balance regulation (*Npy*, *Agrp*, *Pomc*, *Cart*). Values represent Pearson's correlation coefficients (*R*), with all correlations being statistically significant (*p* < 0.001).

## Data Availability

The data that support the findings of this study are available in the Materials and Methods, Results, and/or Supporting Information of this article.
