# Supplementary material for: Early‐Life Betaine Supplementation Drives Sex‐Specific Metabolic Programming in Rats, Affecting FGF21 Signaling and Energy Balance
Source: FASEB J. 2026 Oct 16;40(20):e72390. doi: 10.1096/fj.202603380R (PMC13674722; doi:10.1096/fj.202603380R)
Supplement: Supplementary file 1 [file FSB2-40-e72390-s001.docx]

**Supplementary material**

***Nutritional information on diets and betaine supplementation***

The control diet (CD) provided 2.82 kcal/g, with 72.4% of calories derived from carbohydrates, 9.9% from fat, and 17.6% from protein. In contrast, the Western diet (WD) had a caloric density of 4.7 kcal/g, consisting of 43.0% carbohydrates, 40.0% fat, and 17.0% protein.

For betaine supplementation, the specific daily doses administered during the 20-day suckling period were 6.5, 13.0, 20.0, 26.0, 32.0, 40.0, 50.0, 55.0, 65.0, 75.0, 80.0, 90.0, 100, 110, 110, 120, 130, 140, 150, and 160 µg. These doses were calculated based on the average betaine concentration in rat milk (1) per lactation day and the estimated daily milk intake during suckling, as reported by Kojima et al. (2).

***Supplementary figures and tables***

**Figure S1.** Experimental design. The experiment followed both male and female offspring. The betaine supplementation was equivalent to twice (2×) the average intake obtained during breastfeeding. Animal distribution: CD-dams (n = 12), WD-dams (n = 12); short-term offspring CD-VEH (12 males, 10 females), CD-BET (13 males, 12 females), WD-VEH (10 males, 10 females), WD-BET (8 males, 9 females); long-term offspring CD-VEH (14 males, 12 females), CD-BET (14 males, 12 females), WD-VEH (11 males, 12 females), WD-BET (13 males, 12 females). Abbreviations: C, control; WD, Western diet; VEH, vehicle; BET, betaine; PND, postnatal day.

**Figure S2.** Hepatic *Fgf21* mRNA levels at postnatal day 22 are negatively correlated with adiposity in offspring. The scatter plot depicts the negative correlation between hepatic *Fgf21* mRNA expression (normalized to housekeeping genes) and body fat percentage in offspring at postnatal day 22. Each dot represents an individual animal. Pearson’s correlation coefficient (R) and significance (p-value) are indicated on the plot.

**Figure S3.** Hepatic *Fgf21* mRNA levels at postnatal day 22 are negatively correlated with circulating leptin levels in offspring. The scatter plot illustrates the negative correlation between hepatic *Fgf21* mRNA expression (normalized to housekeeping genes) and circulating leptin levels in offspring at postnatal day 22. Each dot represents an individual animal. Pearson’s correlation coefficient (R) and significance (p-value) are indicated on the plot.

**Figure S4.** The hepatic expression of *Srebp1* and *Scd1* is positively correlated with circulating triglyceride (TG) levels in offspring at 7 months of age. The scatter plots show the positive correlation between circulating TG levels and hepatic mRNA expression (normalized to housekeeping genes) of Srebp1 (A) or Scd1 (B) in offspring at 7 months of age. Each dot represents an individual animal. Pearson’s correlation coefficient (R) and significance (p-value) are shown on each plot.

**Table S1.** Correlation between hypothalamic gene expression levels. The table shows the correlations between *Drd2* and *Fgfr1* expression (mRNA), as well as their correlations with the expression of neuropeptides involved in energy balance regulation (*Npy*, *Agrp*, *Pomc*, *Cart*). Values represent Pearson’s correlation coefficients (R), with all correlations being statistically significant (p < 0.001).

| **Variable 1** | **Variable 2** | **Pearson's correlation coefficient** | ***p*-value** |
| --- | --- | --- | --- |
| *Drd2* | *Fgfr1* | 0.84 | <0.001 |
|  | *Npy* | 0.65 | <0.001 |
|  | *Agrp* | 0.58 | <0.001 |
|  | *Pomc* | 0.76 | <0.001 |
|  | *Cart* | 0.71 | <0.001 |
| *Fgfr1* | *Npy* | 0.62 | <0.001 |
|  | *Agrp* | 0.44 | <0.001 |
|  | *Pomc* | 0.67 | <0.001 |
|  | *Cart* | 0.63 | <0.001 |

**References**

1.	Martín-Chamorro, R. A., Pomar, C. A., Palou, A., Picó, C., and Rodríguez, A. M. (2025) Impact of Western diet on milk miRNAs and target genes in offspring adipose tissue: modulation by betaine during suckling. *Obesity (Silver Spring)* **33**, 732-742

2.	Kojima, T., Nishimura, M., Yajima, T., Kuwata, T., Suzuki, Y., Goda, T., Takase, S., and Harada, E. (1998) Effect of intermittent feeding on the development of disaccharidase activities in artificially reared rat pups. *Comp Biochem Physiol A Mol Integr Physiol* **121**, 289-297
